# Effect of Medicine-Separated Moxibustion on Navel Combined with Mind-Regulating Acupuncture on Curative Effect and Quality of Life in Patients with Allergic Rhinitis

**DOI:** 10.1155/2022/4093891

**Published:** 2022-05-31

**Authors:** Tenggang Ma, Hongyu Zhang, Renzhong Wang

**Affiliations:** ^1^School of Traditional Chinese Medicine, Shandong University of Traditional Chinese Medicine, Jinan 250000, China; ^2^First Clinical School of Medicine, Shandong University of Traditional Chinese Medicine, Jinan 250000, China; ^3^Department of Otolaryngology, The First Affiliated Hospital of Shandong University of Traditional Chinese Medicine, Jinan 250000, China

## Abstract

**Objective:**

To explore the effect of medicine-separated moxibustion combined with mind-regulating acupuncture on the efficacy and quality of life of patients with allergic rhinitis (AR).

**Methods:**

Sixty patients with AR cured from February 2019 to April 2021 were enrolled in our hospital. The patients were arbitrarily assigned into control and study groups. The former group was treated with herbal moxibustion on the navel, while the latter group was treated with herbal moxibustion on the navel combined with mind-regulating acupuncture. The curative effect, TNSS score, TNNSS score, RQLQ score, and the incidence of adverse events were compared.

**Results:**

Compared with the two groups, the study group was markedly effective in 24 cases, effective in 5 cases, and ineffective in 1 case, with an effective rate of 100.00%, while the control group was markedly effective in 13 cases, effective in 10 cases, and ineffective in 7 cases, with an effective rate of 100.00%. The curative effect of the study group was better compared to the control group (*P* < 0.05). There was no remarkable difference in TNSS score before treatment (*P* > 0.05). After treatment, the TNSS scores of patients decreased. Compared with the control group, the TNSS score of the study group was lower than that of the control group at 2 weeks, 4 weeks and during the follow-up. No remarkable difference appeared in the TNNSS score before treatment (*P* > 0.05). After treatment, the TNNSS scores of patients were decreased. Comparing the two groups, the TNNSS scores of the study group were lower compared to the control group after 2 weeks of treatment, 4 weeks of treatment and during the follow-up period (*P* < 0.05). There was no remarkable difference in the RQLQ score before treatment (*P* > 0.05). After treatment, the RQLQ scores of patients were decreased. Comparing the two groups, the RQLQ scores of the study group were lower compared to the control group at 2 weeks, 4 weeks after treatment and during the follow-up period (*P* < 0.05). In terms of the incidence of adverse events, the incidence of adverse events such as subcutaneous hematoma, bruising, and induration in the study group was lower compared to the control group (*P* < 0.05).

**Conclusion:**

Medicine-separated moxibustion was combined with mind-regulating acupuncture when treating AR. There were differences in clinical efficacy, single-symptom score, and TNSS, TNNSS, and RQLQ scores, which verified the clinical efficacy of medicine-separated moxibustion combined with mind-regulating acupuncture when treating AR, and expounded the mechanism of medicine-separated moxibustion combined with mind-regulating acupuncture when treating AR. In the meantime, it shows that the umbilical method of medicine-separated moxibustion combined with mind-regulating acupuncture when treating AR has the advantages of definite short-term effect, long-term effect, safe and simple operation, and no adverse reactions, which is worthy of clinical application.

## 1. Introduction

Allergic rhinitis (AR) is a common nasal disease in clinic, which is characterized by sneezing, runny nose, stuffy nose and itching, which brings a lot of inconvenience to patients' study, work and life, and seriously affects their mental state [1]. At present, AR has become a health concern in various regions. With the development of economy and the change of people's lifestyle, the incidence rate is on the rise. According to statistics, the global incidence rate is between 10.1% and 25.4%. In China, the number of patients with AR is increasing year by year, the incidence rate is as high as 8.7%–24.1% and 40% in some areas [1, 2]. AR is assigned into persistent and intermittent according to duration and mild and moderate to severe according to severity. If there is no timely and effective treatment, it is likely to induce other allergic diseases and cause physical discomfort. At present, the pathogenesis of AR in modern medicine is not completely clear. It is recognized that the pathogenesis is a series of immune responses mediated by IgE and combined with a variety of immune active cells [2]. The incidence of AR is related to many factors. The common treatment methods of Western medicine are hormone therapy, antihistamine therapy, and immunotherapy, which can obtain certain curative effect, but it has some side effects, drug dependence, and easy recurrence [3]. The long-term effect is not good, and there is a certain degree of nephrotoxicity; the disease cannot be cured as a whole [4]. Therefore, in order to make the curative effect lasting and effective, actively looking for the exact treatment plan has far-reaching social significance.

AR belongs to the category of “rhinorrhea” in traditional Chinese medicine (TCM) [5]. The nose, lung orifice, and nose disease should be responsible for the lung, so its lesion site is mainly in the lung, but also because of the five elements, and the five internal organs are closely related to the spleen and kidney [6]. Therefore, the syndrome types of TCM often include deficiency and cold in the lung, deficiency of spleen, and spleen and deficiency of kidney yang, and the treatment is mainly to support vital qi, invigorate the spleen and warm the kidney, and clear the lung and the orifices. TCM has its unique advantages when treating AR. Over the years, clinicians have adopted TCM, acupuncture, natural moxibustion, and other methods to treat this disease and achieved remarkable results, and there were no serious adverse reactions, being safe and reliable [7]. Medicine-partitioned moxibustion on the navel is a characteristic external treatment of traditional Chinese medicine. In the late 1980s, China began to study navel therapy for diarrhea, irritable bowel syndrome, primary dysmenorrhea, infertility, and other diseases [8]. Medicine-separated moxibustion navel therapy was used to treat many patients with AR and achieved relatively satisfactory results [9].

In recent years, with the increase of the incidence of AR, the psychological problems of patients with AR have been paid more attention, which mainly includes three aspects: the first is the discomfort caused by the symptoms themselves, the second is the patients' worries about the treatment methods, drug side effects, and prognosis of the disease, and the third is the time and economic cost of treating the disease [10]. The resulting irritability, anxiety, insomnia, and nervousness may decrease the function of the immune system and aggravate the disease. Somatic symptoms and psychological factors influence each other and become one of the factors affecting the clinical efficacy of AR. The concept of the integration of form and spirit is one of the important contents of TCM [11]. At present, there are two broad views on “God.” One is the internal function of the life activities of the human body, and the other is the mental activities of the human body, which can also reflect the internal functional state of the body [12]. Therefore, Tiaoshen plays an important role when treating AR. In the Lingshu Sutra, there has long been the description of “roughly keeping the shape, keeping the spirit on the top” and “using the needle without forgetting its spirit.” Paying attention to regulating the mind is also an important content of acupuncture, which runs through all stages before and after treatment, paying attention to the four diagnosis components and examination of the mind before treatment; selecting the acupoints to regulate the mind, the doctor “must be one of his spirits,” and the patient will keep his mind after treatment. After treatment, patients pay attention to the doctor's orders to nourish the mind. Doctors regulate the mind and protect vital qi [13]. Based on this, this study focuses on the effect of medicine-separated moxibustion combined with mind-regulating acupuncture on the efficacy and quality of life of patients with AR.

## 2. Patients and Methods

### 2.1. Normal Information

Sixty patients with AR cured from February 2019 to April 2021 were enrolled in our hospital. The patients were arbitrarily assigned into control and study groups. The former group was treated with herbal moxibustion on the navel, while the latter group was treated with herbal moxibustion on the navel combined with mind-regulating acupuncture. In the control group, the age was 21–69 years old, with an average of (40.91 ± 3.55) years, including 16 males and 14 females. In the study group, the age was 44–76 years old, with an average of (40.34 ± 3.64) years, including 13 males and 17 females. There exhibited no statistical significance in the general data. This study was permitted by the Medical Ethics Association of our hospital, and all patients noticed informed consent.

*Diagnostic Criteria*. The diagnostic criteria of Western medicine refer to the diagnostic criteria of AR in the guidelines for the diagnosis and treatment of AR (Tianjin, 2015) [14]: (1) Clinical symptoms: sneezing, runny nose, stuffy nose, and nasal itching occurring twice or more; the cumulative time of symptom attack within a day is longer than 60 min, and symptoms such as eye itching, tears, headache, and ear itching may occur during the attack; (2) physical signs: the nasal mucosa was light gray or pale, edema, and clear nose accumulated in the nasal cavity. The diagnostic criteria of TCM refer to the diagnostic basis of nasal dysphagia in the guidelines for diagnosis and treatment of Common Diseases in Otorhinolaryngology [15]: (1) Medical history: the relationship between the onset of the disease and irritants such as seasons, environmental changes, and inhalation; whether the patient has a family history and a history of allergies; (2) symptoms: sudden and recurrent sneezing, runny nose, stuffy nose, and itching are the main symptoms, such as itching, tears, headache; (3) examination: the nasal mucosa is pale and edema, especially in the inferior turbinate, and a large amount of water-like nasal discharge accumulates in the nasal cavity.

Inclusion criteria: (1) those who meet the diagnostic criteria of Western medicine, TCM, and syndrome of AR mentioned above; (2) aged between 18 and 70 years old; (3) did not take drugs and other related treatment within 2 weeks before the trial; and (4) voluntarily joined the subject and signed the informed consent form.

Exclusion criteria: Those who meet the following 1 item can be excluded: (1) patients with acute and chronic paranasal sinusitis, bronchitis, pneumonia, and other respiratory related diseases; (2) patients with asthma and other allergic diseases; (3) organic lesions in the nasal cavity, or those who have undergone nasal surgery; (4) patients with mental disorders; and (5) pregnant women and breast-feeding patients.

Elimination standard: Those who meet the following items should be excluded: (1) subjects with poor compliance, unable to return on time, unable to cooperate with the treatment of this trial; (2) the use of other treatment methods that may result in intervention, such as the use of drugs; and (3) incomplete data that affect the evaluator of the curative effect.

Shedding standard: (1) the patient withdrew himself; (2) the treatment was interrupted for unknown reasons.

### 2.2. Treatment Methods

The control group received herbal moxibustion on the umbilicus; the drugs were aconite, cinnamon, salt eucommia, psoralen, raw astragalus, raw ephedra, asarum, Xinyi, goose not eating grass, and borneol according to 3 : 3:3 : 3:3 : 2:1 : 2:2 : 1 ratio, pulverize it with an ultrafine pulverizer, sieve to get the fine powder, and store the powder in a sealed container for later use. Method of operation: first mix the flour, knead the dough into a cylindrical doughnut with a base diameter of about 6 cm and a height of about 2 cm, squeeze one side of the edge around 1 cm, and leave the left and right face of 1 cm in the middle. The Schilling patient lies flat on the treatment bed and fully exposes the moxibustion site. After routine disinfection, the doughnut is placed on the navel, the hole of the doughnut is aligned with the Shenque acupoint, and the hole towel is placed on the patient's abdomen to keep warm. Then take appropriate amount of medicine powder (about 3G) to fill the doughnut hole and gently press, put the premade conical moxa (base diameter about 2.5 cm, high about 2 cm) on the medicine powder, ignite the moxa, and replace the next strong when the moxa is completely burned out, and apply moxibustion for 1.5 hours continuously. At the end of moxibustion, acupoint stickers were used to seal the umbilical herbal medicine; remove it 8 hours later, and clean the residual drugs in the umbilical cord. Course of treatment: once every 3 days, twice a week, 4 weeks as a course of treatment, and the curative effect was evaluated after one course of treatment.

The study group was treated with herb-partitioned moxibustion on the umbilicus combined with mind-regulating acupuncture: Baihui, Yintang, Yingxiang, Hegu, Taichong, Dazhui, Feishu, Ganshu, Pishu, and Shenshu. Operation: patients first take prone position, after routine disinfection of the local skin surface of acupoints with 75% alcohol, select 1.5-inch filiform needle, select acupoints according to standard acupoints, acupuncture Dazhui, Feishu, Pishu, Ganshu, and Shenshu, each point is given twirling tonifying method for 30s, retaining needle 20 min. Then take supine position, such as before disinfection, acupuncture Baihui, Yintang, Yingxiang, Taichong, Hegu, Baihui, Yintang group 1, and bilateral Yingxiang group 1; each group received electroacupuncture, enrolled continuous wave, the frequency was 2 Hz, each point was given the method of tonifying and relieving diarrhea for 30 seconds, the stimulation intensity was not caused by patient discomfort, and needle 20 min was retained. The patients were treated 3 times a week for 6 weeks for a total of 18 times.

### 2.3. Observation Index

#### 2.3.1. Evaluation of Curative Effect

According to “principles and recommended programs for diagnosis and treatment of AR” (Lanzhou, 2004), the AR curative effect was judged [16]. Before and after treatment and during follow-up, the symptoms and signs of the patients were recorded, and the curative effect was evaluated according to the sum of symptoms and signs before and after treatment. Efficacy index (*n*) = [(total score before treatment-total score after treatment)/total score before treatment] × 100% < 26% is invalid, 26% ≤ *n* < 66% is effective, and ≥ 66% is remarkable.

#### 2.3.2. Total Score of Nasal Symptoms (TNSS)

The scale scores from four aspects: stuffy nose, runny nose, itching, and sneezing, each of which contains 5 different grades, 0: asymptomatic; 1: mild; 2: moderate; 3: severe; and 4: very heavy. Patients were scored based on the severity of individual symptoms and a cumulative total of 16 points was calculated.

#### 2.3.3. Evaluation Form of Concomitant Symptoms of Rhinitis

Evaluation of concomitant symptoms of rhinitis (Total Non-Nasal Symptom Score, TNNSS) [17]. The scale includes 5 items: (1) snot flows through the pharynx; (2) tears; (3) nasal or eye itching; (4) nasal or oral maxillary pain; and (5) headache. Each item was assigned into two grades: symptomatic and asymptomatic, which were scored as 1 and 0, respectively, and the highest total score was 5.

#### 2.3.4. Quality of Life Questionnaire for Patients with Nasal Conjunctivitis (RQLQ)

This questionnaire includes (1) activity; (2) sleep; (3) nonnasal/eye symptoms; (4) practical problems; (5) nasal symptoms; (6) eye symptoms; and (7) emotion. A total of 7 parts involved 28 questions. Except for the activity part, the score of each question in the other six parts includes 7 grades—0: no trouble; 1: almost no trouble; 2: some trouble; 3: moderate distress; 4: very troubled; 5: very troubled; and 6: extremely troubled. There is one more level in the activity section, and a score of 9 indicates that no activity has been done. The patients were scored according to their personal conditions, and the cumulative total score was calculated, with a maximum of 177 points [18].

### 2.4. Adverse Events

Recording and dealing with adverse events: closely observe the status of patients in the course of treatment, detect patients' adverse reactions in time and make a correct judgment of their prognosis, and make corresponding records. In addition, in the course of treatment, if other treatment is necessary for special reasons, detailed records should be made.

### 2.5. Statistical Analysis

Using SPSS21.0 statistical software, before statistical analysis, the measurement data were examined by normal distribution and variance homogeneity analysis to meet the requirements of normal distribution or approximate normal distribution, presented as $\bar{x}$  ± *s*, and repeated measurement data were analyzed by repeated measurement analysis of variance. *t*-test was adopted to compare, *n* (%) was adopted as an example to represent the counting data, and *χ*^2^ test was adopted to indicate that the difference exhibited statistically remarkable (*P* < 0.05).

## 3. Results

### 3.1. Comparison of Curative Effect

**Figure 1:**
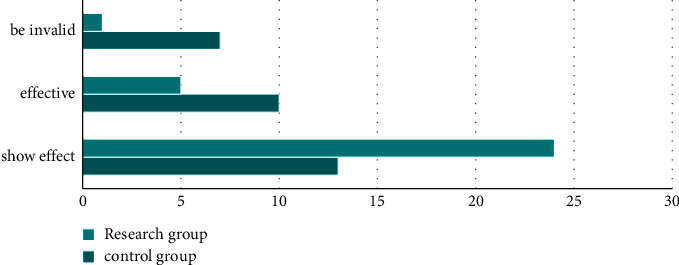
Comparison of curative effect between two groups.

The curative effect of the study group was remarkably effective in 24 cases, effective in 5 cases, and ineffective in 1 case, with an effective rate of 100.00%. In the control group, 13 cases were markedly effective, 10 cases were effective, and 7 cases were ineffective, with an effective rate of 100.00%. The curative effect of the study group was better (*P* < 0.05). All the data results are indicated in Figure 1.

### 3.2. TNSS Score Comparison

There was no remarkable difference in TNSS score before treatment (*P* > 0.05). After treatment, the TNSS scores of patients were decreased. Compared with the two groups, the TNSS scores of the study group were lower at 2 weeks, 4 weeks and during the follow-up period (*P* < 0.05). All data results are indicated in Table 1.

### 3.3. TNNSS Score Comparison

There was no remarkable difference in TNNSS score before treatment (*P* > 0.05). After treatment, the TNNSS scores of patients decreased. Comparing the two groups, the TNNSS scores of the study group were lower at 2 weeks, 4 weeks of treatment and during the follow-up period (*P* < 0.05). All data results are indicated in Table 2.

### 3.4. RQLQ Score Comparison

There was no remarkable difference in RQLQ score before treatment (*P* > 0.05). After treatment, the RQLQ scores of patients were decreased (*P* < 0.05). Comparing the two groups, the RQLQ scores of the study group were lower at 2 weeks, 4 weeks after treatment and during the follow-up period (*P* < 0.05). All the data results are indicated in Table 3.

### 3.5. Comparison of Incidence of Adverse Events

**Figure 2:**
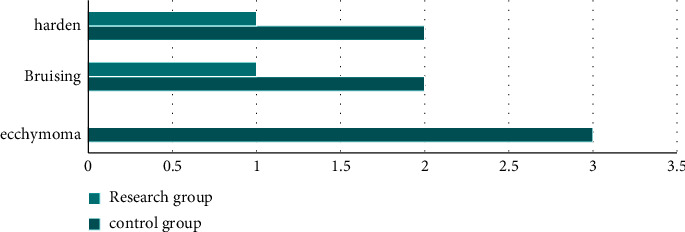
Incidence of adverse reactions in two groups.

With regard to the incidence of adverse events, the incidence of subcutaneous hematoma, stasis, induration, and other adverse events in the study group was remarkably lower (*P* < 0.05). All the data results are indicated in Figure 2.

## 4. Discussion

Allergic rhinitis (AR) belongs to the category of “rhinitis” in TCM. Ancient books are often named “sneeze” according to its clinical manifestations [19]. Nose sneeze is also known as “sneeze” and “spider sneeze.” In Shuowenjiezi, it means “cold nose.” In the “release of the name,” “stuffy nose is said to be stuffy for a long time.” The symptoms of Bixun are characterized by stuffy nose and runny nose. It is recorded in the Book of Rites of the Western Zhou Dynasty that the autumn goes to summer and the country is flooded [17]. Min duo sneeze is the earliest description of its etiology and pathogenesis. The Internal Classic of the Yellow Emperor in the Spring and Autumn and Warring States Period put forward the name of the disease, “the kidney is a sneeze, and the Yangming place is a sneeze.” This paper discusses the correlation with Yangming pulse, Taiyin pulse, and five movements and six qi [18]. Husband Jin Jin Jin, heat is dry, cold is overflowing, can't take it by oneself [20]. Lung qi is in the nose, its internal organs are cold, cold into the nose, so that Jinjie cannot collect itself. “In the Song Dynasty, Yan Yong and in the Yan Shi Jisheng Fang said: “the husband's nose is the condition of the lungs.” If the seven emotions are depressed. It is also a disease. It is clear runny [21]. If the wind-cold in the nose is multiplied by the wind-cold in the lung, the yang meridian will be unfavorable, resulting in congestion or clear and runny nose. During the Jin and Yuan dynasties, Liu Hejian explained in “Su Meng Xuanji original Disease Volume 1”: “the nose is clear and sneezing, and the breath in the nose is sprayed on the sound because of itching.” [22]. During the Ming and Qing dynasties, most doctors took “nose malaise” as the name of the disease, as recorded in Li Shizhen's Compendium of Materia Medica in the Ming Dynasty, “nose diarrhea, runny nose, brain cold, heat inside” [23]. Although different generations of physicians have not unified the title of rhinorrhea, the expression of clinical symptoms is relatively consistent. Through the description of the symptoms of rhinorrhea, it provides a relatively clear historical context for the historical development of rhinorrhea [23]. Until the 1980s, the national medical colleges and universities tried out the textbook Otorhinolaryngology of TCM with the correct name of “nasal shooting.” Since then, it clearly corresponds “Bixun” to AR [24].

The etiology of rhinorrhea is complicated, which is mainly caused by physical factors, diet fatigue, feeling external evil, and emotional depression. Deficiency syndromes are mostly yang deficiency of lung, spleen, and kidney, loss of osmosis of nose, syndrome of external pathogen invading lung, loss of lung or depression, loss of liver and leakage, and obstruction of nasal orifices [25]. Conclusively, it mainly includes the following aspects: lung deficiency and nonsolid, wind-cold invasion: lung main qi, division of breathing, in the external combination of fur and delicate dirt, easy to feel the invasion of external evil. The wind is the length of all diseases, when the wind-cold combined with evil attack the lung, resulting in the loss of lung, lung qi is disadvantageous; lung opening in the nose, lung qi is disadvantageous, then the nose is blocked; loss of lung qi, lung deficiency is not solid; if qi does not absorb fluid, then the nose is clear. “The lung qi passes through the nose, and the internal organs are cold, and the cold is multiplied by the qi in the nose, so the records in the theory of pathogenic factors directly show the symptoms of runny nose after the lung is attacked by wind and cold [26]. In Jingyue Encyclopedia, “for those who suffer from cold and stuffy nose, the meridians are congested and sneeze more often.” This syndrome is mostly in the Sun Meridian, which emphasizes the symptoms of sneezing and runny nose caused by wind-cold invasion. Spleen deficiency does not move, Qingyang does not rise: the spleen dominates the movement, the main ascending clear and descending turbid, the spleen likes irritability and dislikes dampness. According to the introduction to Medicine, “the nose is the way of clearing qi in and out of the stomach.” The clearness of the nose depends on the nourishment of the temper [27]. The normal metabolism of water is “drinking the stomach, overflowing the essence, infusing the spleen, dispersing the essence in the spleen, returning to the lung, regulating water, infusing the bladder, four cloths of water and essence, and the five liquids are juxtaposed.” When living in wetlands for a long time, overwork, unregulated diet, and other reasons lead to spleen loss of health, dampness, and pathogenic endogenesis, spleen is not clear, nasal orifices are not nourished, and nasal congestion is disadvantageous; soil does not give birth to gold, lung qi deficiency, lung loss, and descending body fluid accumulation; it is called nasal dysentery [27, 28]. Loss of the liver and osmosis of the nose: the liver reaches the level of the liver, which is the viscera of the wind wood of Jueyin, and its meridians “go up into stubborn pharynx” and pass through the nasopharynx. If the emotion is excessive, it leads to the disorder of the liver, the imbalance of qi, and the loss of osmosis of the nose; “liver left ascends and lung descends right” means: Liver failure, Qi, and blood disorders, leading to lung disorders, poor nostrils, nasal congestion, and runny nose [28]. Deficiency of kidney qi, evaporation has no right: the kidney dominates the absorption of qi, which is the root of yang qi. The kidney yang is sufficient, the lung is warm, and the intake of qi is normal. With the deficiency of kidney qi, deficiency of kidney yang, loss of warmth of the lungs, invasion of water, and loss of the lungs, the nose is clear. “Su Wen Xuanming Five Qi Theory” records as follows: “At the age of sixty, yin phlegm, qi greatly declines, the nine orifices are unfavorable, the lower is empty and the upper is solid, and tears come out.” Loss declared surrender, sneezing and tears appeared. In addition, there is the theory of “lurking evil in the interior, there is stagnant heat inside,” which holds that it is caused by endogenous or external evil that lurks in the viscera, meridians, and collaterals, and then occurs due to external causes; the evil qi turns into heat for a long time, or excessive diet and depression, resulting in stagnant heat, so Evil qi will turn into heat for a long time, which will lead to stagnation of food accumulation, so the disease is hot on the surface, but actually cold, which is commonly called lung cold [29]. However, when I saw the cold room, I thought it was very cold. I did not know that the skin was cold and dense, and the heat was depressed and the disease became more serious. In addition, there are “latent toxin theory” and “potential dryness theory” to explain the relationship between exogenous and endogenous toxins, exogenous and endogenous dryness, and nasal symptoms, such as nasal congestion, itching, runny nose, and sneezing, respectively [30, 31].

Medicine-separated moxibustion on the navel, also known as the steaming navel method, refers to a method of filling the navel after grinding the medicine into powder, placing a doughnut in the middle of the navel, and moxibustion in the middle of the navel, which is a kind of external treatment of TCM. It is a treatment method that integrates effectiveness, safety, and comfort and has been widely adopted in the long-term clinical practice. Wu Shiji in the Qing Dynasty said: “the principle of external treatment is the principle of internal treatment, and the medicine of external treatment is the medicine of internal treatment.” The navel method of medicine-separated moxibustion is effective mainly through meridians and acupoints, drug penetration, and absorption and moxibustion [32]. The navel is the Shenque acupoint, which is known as the vital pedicle, the former life gate, and the air house. Its importance has long been recorded in ancient literature. “Sixteen difficulties” clearly points out that the diseases of the five internal organs can be judged according to the tenderness of the corresponding parts around the umbilical cord. “Dysmenorrhea 66 is difficult to say:” those who move qi between the kidneys under the navel, human life. “From the meridian point of view, Shenque belongs to Ren meridian acupoint, which is closely related to Chong, Ren, du and belt. The navel communicates with the internal and external organs through meridians, meridians and tendons, and is the general pivot of meridians and collaterals. Stimulating this acupoint can play the effect of regulating the qi and blood of the whole-body meridians. From the point of view of modern medicine, the umbilical cord is the latest closure of the abdominal wall in the stage of embryonic development, and it is the weak area of the anterior abdominal wall, its subcutaneous stratum corneum is the thinnest, there is no accumulation of adipose tissue in the deep surface, and the skin and umbilical fascia can be directly connected with the mural peritoneum. In the meantime, there is a periumbilical venous network in the umbilical part, which is directly connected with the hepatic portal vein, upward with superior abdominal wall arteries and veins, downward with inferior abdominal wall arteries and veins, superficial abdominal wall arteries and veins, and rich blood supply; the umbilical cord is close to the abdominal pelvic cavity, and the lower part is the digestive tract system. There is also a corresponding relationship with the kidney, and there are a large number of autonomic nerve plexuses nearby, which can dominate the organs and blood vessels in the abdominal and pelvic cavity [33]. Studies have indicated that stimulation of Shenque acupoint will stimulate the nerve endings of the umbilical cord and then promote the regulation of nerve and humoral immunity, accelerate blood circulation, promote metabolism, and enhance immune ability [34]. From the analysis of the umbilical structure, it is considered that the umbilical permeability is the best, and it can better absorb the drug effect compared with oral medicine. In the meantime, umbilical medication avoids the adverse reactions caused by oral drugs to the body [35]. Therefore, medicine-separated moxibustion navel therapy is a comprehensive therapy integrating meridians, acupoints, TCM, and moxibustion, adhering to the overall concept, applying both symptoms and root causes, and has the advantages of definite short-term effect, long-term effect, high cure rate, safety, and no adverse reactions. Medicine-partitioned moxibustion on the umbilicus is suitable for patients with weak stomach, drug intolerance, digestion and absorption disorders, fear of acupuncture, and side effects of western medicine. Medicine-separated moxibustion navel method is an effective, safe, and comfortable treatment method, which can enhance the constitution of kidney-yang deficiency, alleviate clinical symptoms, and improve the life treatment of patients [36].

In recent years, there are more studies on the relationship between mental state and AR symptoms of AR patients, and the clinical research on the treatment of AR from the perspective of “mind regulation” is also abundant. Acupuncture is one of the main external treatment methods of TCM when treating AR, which has the characteristics of clear curative effect, little side effect, convenient use, and easy operation, so it is widely adopted in clinic. There are many acupuncture points and methods, including mind-regulating acupuncture, sensation transmission acupuncture, Yi Shou acupuncture, warming Yang acupuncture, Xuanfei Tongqiao acupuncture, Tongyuan acupuncture, improved acupuncture, and acupuncture at specific acupoints Xinwu. Professor Zhao Jiping's team put forward the method of mind-regulating acupuncture combined with clinical practice. Baihui, Yintang, Yingxiang, Hegu, Taichong, Dazhui, Feishu, Pishu, and Shenshu were enrolled for treatment in combination with brain-regulating, mind-regulating, visceral mind-regulating, qi-regulating machine, Changqing emotion, and other aspects. It was found that after 4 weeks of treatment, the curative effect was similar to that of oral cetirizine hydrochloride tablets. In the follow-up clinical study, it was found that the long-term effect of mind-regulating acupuncture was better [37]. David et al. choose Shenmen acupoint to recuperate the mind in the clinical treatment of AR to make the curative effect more stable and the recurrence rate lower [38]. Marcin believes that Bi syndrome is a symptom of deficiency, that is, deficiency of the lungs, spleen, and kidney, which is actually the invasion of external pathogens such as wind and cold [39]. During treatment, attention is paid to regulating yin and yang and putting forward “Tongyuan therapy”; that is, “Tongdu Tiaoshen” selects Governor Shang Xing, Baihui, Yintang, Dazhui, or five Zang back Shu points to regulate mind and spirit and selects Ren Meridian acupoints Qihai, and Guanyuan, advocating syndrome differentiation and acupuncture combined with medicine. Nosulya and Kim put forward the idea of “regulating the mind with the spirit and distracting the mind with the needle,” paying attention to the regulating spirit and brain spirit, selecting Baihui, Yintang, and Neiguan acupoints [40]. Mazurek Jacek and Henneberger Paul selected Dazhui, Shangyingxiang, Yingxiang, Sibai, Shangxing, Yintang, Shize, Lieqian, and Hegu points from “shape and spirit homology” to treat persistent moderate and severe AR that has the same curative effect as Western medicine, and meanwhile, the long-term effect is better [41]. In a clinical trial of 221 patients in a multicenter case series study of acupuncture on the sphenopalatine ganglion for the treatment of AR, it was found that from the 2nd week to the 8th week of acupuncture, the nasal symptom scale (TNSS) and rhinitis were accompanying symptom scale (TNNSS) score that continued to decrease, RQLQ score continued to decrease until the 8th week, the difference was statistically remarkable, and one of them had swelling of the cheek, but after symptomatic treatment, the symptoms disappeared after 3 days [42].

In summary, medicine-separated moxibustion is combined with mind-regulating acupuncture when treating AR; there were differences in clinical efficacy, individual symptom scores, TNSS, TNNSS, and RQLQ scores, which verified the clinical efficacy of medicine-separated moxibustion combined with mind-regulating acupuncture when treating AR and expounded the mechanism of medicine-separated moxibustion combined with mind-regulating acupuncture when treating AR. At the same time, it is shown that medicine-partitioned moxibustion combined with mind-regulating acupuncture in the treatment of AR has the advantages of precise short-term curative effect, long-term curative effect, safe and simple operation, and no adverse reactions, and is worthy of clinical application [43].

## Figures and Tables

**Table 1:** Comparison of TNSS scores of patients [$\bar{x}$ ±*s*, points].

Group	N	Before treatment	Treatment for 2 weeks	Treatment for 4 weeks	Follow-up period
C group	30	9.94 ± 2.45	6.46 ± 1.45	4.59 ± 1.23	4.53 ± 1.77
R group	30	9.93 ± 2.47	5.01 ± 1.55	4.03 ± 0.11	3.58 ± 0.67
T		0.015	3.741	2.483	2.749
P		＞0.05	＜0.01	＜0.01	＜0.01

**Table 2:** Comparison of TNNSS scores [$\bar{x}$ ±*s*, points].

Group	N	Before treatment	Treatment for 2 weeks	Treatment for 4 weeks	Follow-up period
C group	30	2.94 ± 1.34	1.79 ± 0.63	1.36 ± 0.15	1.09 ± 0.33
R group	30	2.90 ± 1.56	1.48 ± 0.14	1.04 ± 0.55	0.74 ± 0.12
T		0.160	2.630	3.074	5.459
P		>0.05	<0.01	<0.01	<0.01

**Table 3:** Comparison of RQLQ scores [$\bar{x}$ ±*s*, points].

Group	N	Before treatment	Treatment for 2 weeks	Treatment for 4 weeks	Follow-up period
C group	30	77.48 ± 10.33	63.48 ± 10.44	49.39 ± 4.66	48.39 ± 4.31
R group	30	77.49 ± 10.45	58.92 ± 9.42	40.95 ± 8.31	38.49 ± 6.34
T		0.003	2.115	4.852	7.037
P		>0.05	<0.01	<0.01	<0.01

## Data Availability

The data used to support this study are included within this paper.
